# Growth dynamics of different half-sib families of *Melia azedarach* Linn

**DOI:** 10.1371/journal.pone.0207121

**Published:** 2018-11-12

**Authors:** Sanjeev Kumar Chauhan, Ashok Kumar Dhakad, Rajni Sharma

**Affiliations:** 1 Department of Forestry and Natural Resources, Punjab Agricultural University, Ludhiana, Punjab, India; 2 Department of Botany, Punjab Agricultural University, Ludhiana, Punjab, India; Technical University in Zvolen, SLOVAKIA

## Abstract

The genetic diversity and growth dynamics of fifty-three half-sib families of eleven provenance sources and one bulk seed mixed population of fast growing forest tree species i.e. *Melia azedarach* were studied at two stand ages *viz*., fourth year (mid-rotation) and eighth year (end-rotation) to determine the selection stage in northern India. Significant variations were reported between and within seed provenances in all growth characters at both rotational ages. The broad sense heritability was higher at mid-rotational age. This revealed the growth is genetically controlled but with the time environmental effects escort the growth pattern. Growth pattern was different at each stand age. Growth is diameter dependent and the pattern was crown growth type. Families maintained their superiority over the time for tree height, basal diameter and diameter at breast height, which indicated a strong potential to identify good performing families for future plantation program. This study concluded that early stage selection is appropriate that later stage selection for all parameters studied except clear bole height that is much influenced by management practice and environment factors also. Neighbor-joining clustering with similarity index revealed that it is not necessary that the families, originated in one region were distributed in one cluster, indicating that families with same geographic origin could have undergone changes for different characters under selection.

## Introduction

Each tree species has its own growth rhythm and reacts to seasonal variations. It has been recognized that there are two extremes in the growth allocation patterns [1] of trees: (i) photosynthetic assimilates are allocated more to plant height growth than to distribution of lateral branches and foliage (height-growth type); (ii) assimilates are allocated more to construction of lateral branches and foliage than to plant height growth (crown growth type). These two types correspond to the successional status of species: early-successional species belong to the height-growth type, and late-successional species to the crown-growth type [2,3] and these both types have their significant role i.e., height growth type in forest trees and crown growth type in fruit trees.

Trees have evolved over the period of time and showed a range of growth patterns. This is not only because of a climatic conditions but also edaphic and topographic conditions. Apart from these, seasonal fluctuations in abiotic and biotic factors may altered the growth pattern and stand structure. Lowman [4] discussed three major patterns i.e., a rapid synchronous annual spurt of growth (e.g. *Toona australis*); continuous growth (e.g. *Dendrocnide excelsa*); and an intermittent pattern of continuous growth in spring and summer, followed by winter (e.g. *Doryphora sassafras*). Only few studies have been documented in tropical hardwood as well as deciduous tree species for growth pattern. Deciduous species exhibit predictable annual changes in growth and development rather than evergreen. But two prominent questions are still unanswered, which factors are mainly responsible for differential patterns of growth dynamics within and between trees? and is selection affected by age or growth period? The breeding programme for growth dynamics in forest trees is less adopted due to long life span and less knowledge of long-term impacts due to environmental change. No quantified studies of growth ecology and differential response of progenies of plus trees in *Melia* have been published yet. Therefore, the present study had been planned to investigate the selection and evaluation at different stand ages for growth characteristics, and growth dynamics in size-structured populations of *Melia azedarach*.

*Melia azedarach* Linn. is increasingly used for plantation over the large area in Indian sub-continent owing to its fast growth, multiple uses and ability to withstand a wide range of climatic and edaphic conditions. It is suitable for alkaline soils, waterlogged conditions, and acidic soil [5]. The species has been identified as a potential alternate pulpwood species [6]. Therefore, it has great potential to aid diversification of poplar and eucalypt agroforestry systems in North-Western states in India in near future. In spite of its multiple uses, *M*. *azedarach* has been a subject of limited research especially with reference to tree improvement. The species is of immense importance but no systematic work has been done on early stage/rotational selection, evaluation and identification of potential genetic resources. We, therefore, undertook the current study to select and analyze potential open pollinated half-sib families by assessing the variability to identify best progenies for use in the future improvement of plantation programs. Though this approach, new genes are not created but rather the best of the available genes are accumulated. Hence, the present study is a step in the direction of early stage selection of good genotypes that can be multiplied to produce quality planting material for distribution to farmers instead of long wait till rotation of the species.

## Material and methods

### Study species

*Melia azedarach* Linn. (Synonyms: *Melia Sempervirens*, *M*. *bukayan*, *M*. *japonica*) commonly known by various names i.e., dek, bakain, beed tree, Persian lilac and pride of India, belongs to the Meliaceae or Mahogany family. It is somewhat deciduous in nature, drought sensitive, fire susceptible, identified by fern-like tripinnate foliage and lilac purple flowers. It flowers in April to June (occasional flowering in December in Northern India). Fruits appear after monsoon and are green, and on ripening fruits, ripen yellow in cold season. Seed (drupe) is 1.5 cms in diameter globose, 5 to 6 celled, 5 seeded (or fewer by abortion); yellow when ripe but subsequently wrinkled. Seed is yellowish brown, very hard, each having a natural perforation through the center. Its bark, fruits, leaves, and wood have insecticidal properties [7]. *Melia azedarach* is a multipurpose tree (MPT) and highly valuable for agroforestry/social forestry/urban forestry plantations [8]. This deciduous species grows well with deep roots and adds a large amount of organic matter to the top soil. Besides improving soil aeration, its roots recycle and enrich the soil nutrients [9].

### Study site and methodology

Fifty-three plus trees of *M*. *azedarach* were selected by following base line method from natural stands and plantations at different geographical provenances of Indian sub-continent (Table 1). No specific permissions were required for these locations from where the selections were made. It is also confirmed that the field studies did not involve endangered or protected species. Open pollinated seeds of all plus tree were collected in the month of December-January including one bulk seed mixed population that was treated as control. Dried depulped fruits were soaked in cold water for two days as pre-sowing germination treatment. Dried fruits were sown about 5 to 8 cm deep in raised seed beds under partial shaded in February month and lightly covered with soil. Seeds germinated within 45–60 days after sowing (DAS) and showed 70–80% germination success uniformly in different sources. The seedlings were pricked out into poly bags after 90 days of sowing and planted out in subsequent rainy season at Punjab Agricultural University Seed Farm, Ladhowal, Punjab (India), 30°58´N and 75°44´E, mean elevation 247 m AMSL, area experiences average annual rainfall of 600 mm. The experimental plantation (54 progenies of plus trees of *Melia azedarach* Linn.) was established in a Randomized Complete Block Design [10]. To determine the genetic variation of the introduced families, test materials were grown together in replicates in well-designed trials under potential irrigated condition. An experiment was designed with eight plants with three replication at 4 × 4 m spacing. Thus, total plants per treatments were twenty-four in three replications. The total area used for the experiment was 27000 sq m, which included boarder rows and space between replications as well.

**Table 1:** Details of plus trees and their provenances of *Melia azedarach* Linn.

Source code	Plus trees	Total number	Provenances	Longitude °E	Latitude °N	Altitude
1	1–4	4	CCS Haryana Agricultural University, Hisar (Haryana)	29° 9'0.97"N	75°42'20.12"E	215 m
2	5–9	5	CSK Himachal Pradesh Agricultural University, Palampur (Himachal Pradesh)	32° 6'0.93"N	76°32'47.23"E	1250 m
3	10–16	7	Dr YS Parmar University of Horticulture and Forestry, Solan (Himachal Pradesh)	31°13'26.09"N	76°58'2.06"E	650 m
4	17–21	5	MPKV Rahuri, Ahmednagar (Maharashtra)	19°20'56.72"N	74°38'45.93"E	510 m
5	22–25	4	Marathwada Agricultural University, Parbhani (Maharashtra)	19°15'2.92"N	76°47'42.67"E	347 m
6	26–40	15	Punjab Agricultural University, Ludhiana (Punjab)	30°54'10.10"N	75°48'31.11"E	247 m
7	41–42	2	Rajendra Agricultural University, Pusa Samastipur (Bihar)	25°59'3.99"N	85°40'28.01"E	052 m
8	43–46	4	Sher-E-Kashmir University of Agriculture Science and Technology, Jammu (Jammu & Kashmir)	32°26'19.66"N	75°10'9.33"E	270 m
9	47–48	2	Tamil Nadu Agricultural University, Mettupalayam (Tamil Nadu)	11°19'25.35"N	76°56'10.39"E	300 m
10	49	1	UHF RHRS, Jacch, Nurpur, Kangra (Himachal Pradesh)	32°16'57.76"N	75°51'44.87"E	643 m
11	50–53	4	University of Agriculture Science, Dharwad (Karnataka)	15°29'28.18"N	74°59'6.60"E	678 m
12	54	1	Control (Bulk seed mixed population)			

### Measurements of growth dynamics

Four trees per replication were measured for tree height (TH), clear bole height (CBH), basal diameter (BD) and diameter at breast height (DBH) at two different age rotations *i*.*e*. mid-rotation (4^th^ year) and rotation age (8^th^ year). Total height (m) of a tree was recorded with the help of graduated bamboo pole from ground level to the top of the main shoot. Clear bole height (m) is the stem height up to first green branch was measured with the help of measuring scale of 4 m height. The basal diameter (cm) was measured 5 cm above the base of the tree with the help of tree caliper. The diameter at breast height (cm) was measured at 1.37m from the ground level with the help of tree caliper.

### Statistical analysis

Statistical analysis was done as per the procedure laid down for Complete Randomized Block Design (CRBD). Statistical analysis was carried out using Proc GLM (SAS Software 9.4, SAS Institute Ltd. U.S.A.) and IBM SPSS statistics software’s. The significance of fixed effects was tested with *F* tests. Variation among half-sib families of the sampled clones was analyzed by analysis of variance (ANOVA) between sites [11] using the following equation:

$$y_{ij}=\mu +\alpha_{i}+\beta_{j}+\alpha \beta_{i(j)}+\varepsilon_{ij}$$ (1)

Where y_*ij*_ is the performance of the tree of *i*^th^ family within the *j*^th^ block, *μ* is the overall mean, *α*_*i*_ is the effect of the family (i = 1,……54), *β*_*j*_ is the effect of the block (i = 1,……3), *αβ*_*i(j)*_ is the random effect of the *i*^th^ family with in *j*^th^ block and *ε*_*ij*_ is the random error.

All characters which showed significant differences between the families were subjected to further estimation of genetic parameters. The coefficient of phenotypic variation (*PCV*) was calculated as per the procedure followed by Hai *et al*. [12]. The broad-sense heritability (*H*^*2*^) was calculated as method followed by Hansen and Roulund [11]. The heritability (broad sense) and expected genetic advance resulting from the selection of five per cent superior individuals was calculated following by Lush [13] and Johnson *et al*. [14].

### Growth modeling

Linear and quadratic regression was carried out by Statistical Product and Service Solutions (SPSS) software. Consider a response variable *Y* that can be predicted by a polynomial function of a regressed variable *T*. We estimated a, the intercept; b, the slope due to *T* in the linear form as under:

$$\begin{array}{l} Y_{i}{=a}_{}\;{+\;\mathrm{bX}}_{i}\;\left(X\;\mathrm{referred}\;\mathrm{tree}\;\mathrm{growth}\;\mathrm{component};\;\mathrm{for}\;\mathrm{the}\;\mathrm{observations}\;i=1,2,\ldots \right)\\ Y_{i}{=\;a+\mathrm{bX}}_{i}{+\mathrm{cX}}_{i}{}^{2} \end{array}$$

## Results and discussion

### Genetic variation and evaluation

**Fig 1:**
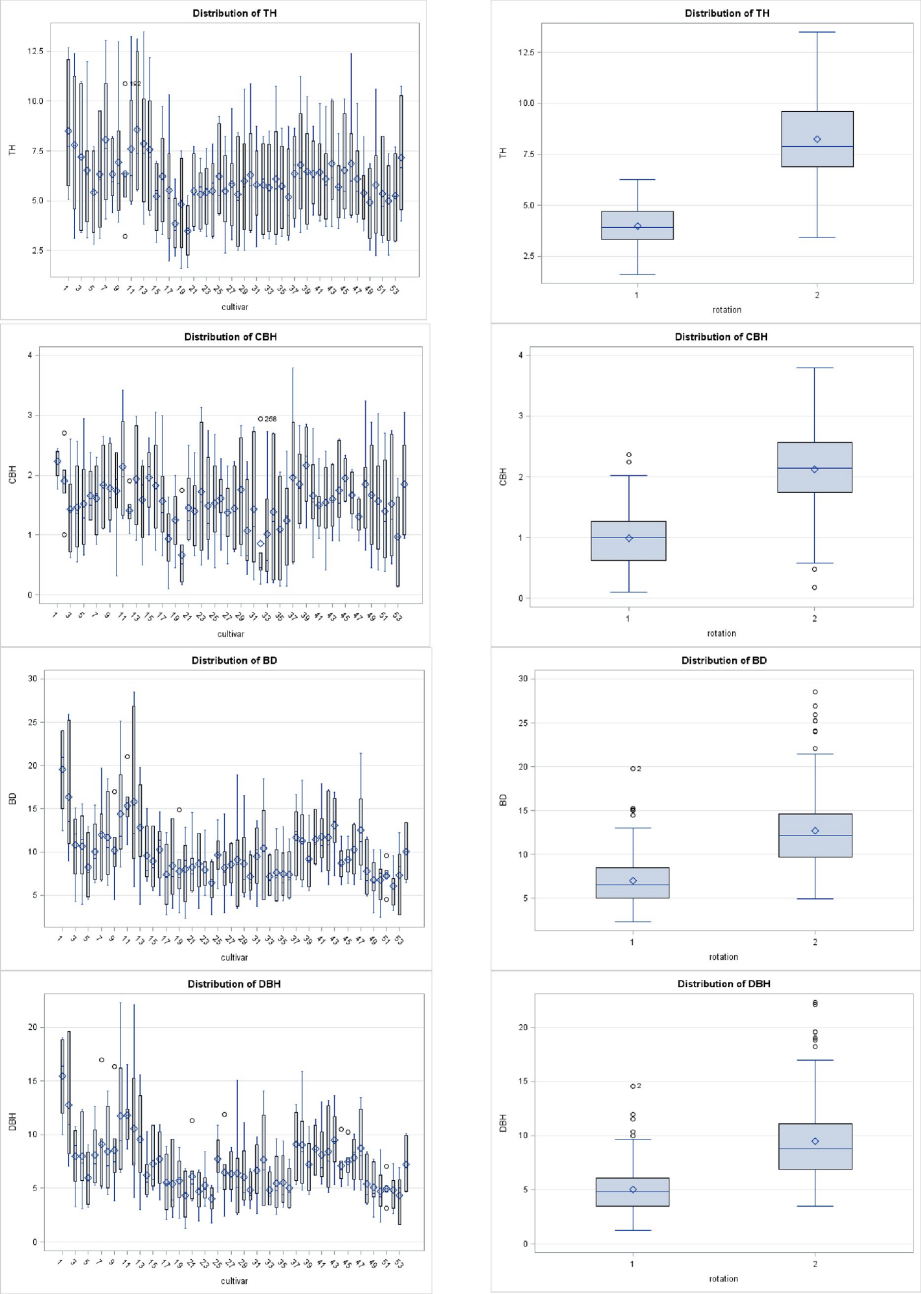
Distribution plots for growth parameters in 54 half-sib families of *M*. *azedarach* Linn. with respect to different age rotation.

**Fig 2:**
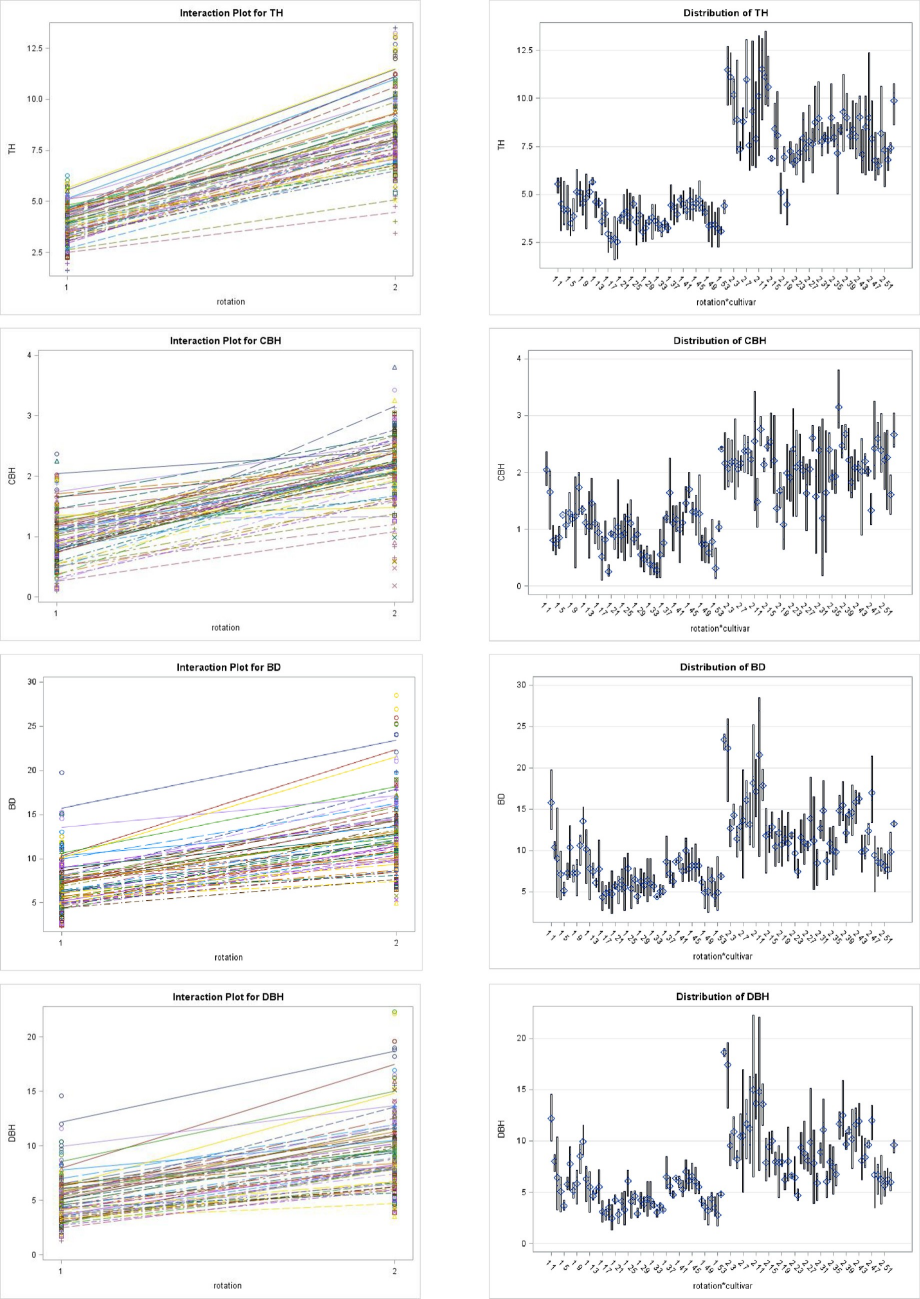
Interaction plots for growth parameters in 54 half-sib families of *M*. *azedarach* Linn. with respect to different age rotations.

Significant differences for each parameter were recorded among between and within families (Table 2), and seed provenances (Table 3) for growth characteristics at two stand ages *i*.*e*. mid-rotation and end-rotation at 5% level of significance (P<0.05). Distribution and interaction of observed tree growth components of fifty-four sources of *M*. *azedarach* at two rotation ages are presented in Figs 1 and 2. In general, the genotypic correlation coefficient values were lower than corresponding phenotypic values. High variance (σ^2^) and heritability (H^2^) were observed at mid-rotation than end-rotation (Table 2). This revealed that environmental factors play a vital role in growth structure at the later stage. The genetic gain was recorded higher for DBH and CBH than TH and BD. Data in Table 2 clearly indicated that TH ranged from 2.51 to 5.64 m and 4.48 to 11.50 m with a variation of 124.4% and 156.7% for the mid-rotation and end-rotation, respectively. CBH ranged from 0.26 to 2.05 m in the fourth year and 1.08 to 3.15 m in the eighth year with a variation of 688.4% and 191.6% for the mid-rotation and end-rotation, respectively. BD ranged from 4.33 to 15.75 cm at mid-rotation with a variation of 263.7% and 7.41 to 23.41 cm with a variation of 215.9% at end-rotation. DBH ranged from 2.46 to 12.17 cm at mid-rotation with a variation of 394.7% and 4.80 to 18.68 cm with a variation of 289.2% at mid-rotation (Table 4).

**Table 2:** Genetic and variation parameters for 54 half-sib families of *Melia azedarach* Linn.

Genetic parameters	TH		CBH		BD		DBH	
	Mid-rotation	End-rotation	Mid-rotation	End-rotation	Mid-rotation	End-rotation	Mid-rotation	End-rotation
LSD_(P<0.05)_	0.941	2.159	0.545	0.860	3.009	5.245	1.839	4.150
SEm	0.333	0.763	0.193	0.304	1.064	1.854	0.650	1.467
Genetic variance (Vg)	0.413	1.615	0.120	0.075	4.073	9.371	3.076	6.656
Phenotypic variance (Vp)	0.745	3.363	0.232	0.353	7.467	19.688	4.345	13.115
GCV	16.088	15.394	34.941	12.927	28.594	24.069	34.826	27.300
PCV	21.613	22.213	48.541	27.960	38.718	34.887	41.388	38.321
Heritability (broad sense)	0.554	0.480	0.518	0.214	0.545	0.476	0.708	0.508
Genetic advance	0.985	1.815	0.514	0.262	3.070	4.351	3.040	3.786
Genetic gain	24.671	21.977	51.813	12.312	43.500	34.207	60.369	40.066

**Table 3:** Variation in different seed provenances* of *Melia azedarach* Linn.

Code	Name of Institution/University and State	TH		CBH		BD		DBH	
		Mid-rotation	End-rotation	Mid-rotation	End-rotation	Mid-rotation	End-rotation	Mid-rotation	End-rotation
1	CCS Haryana Agricultural University, Hisar (Haryana)	4.61 (2)	10.41(1)	1.31 (2)	2.21 (5)	10.56 (1)	18.18 (1)	7.92 (1)	14.15 (1)
2	CSK Himachal Pradesh Agricultural University, Palampur (Himachal Pradesh)	4.43 (4)	8.79 (4)	1.13 (5)	2.23 (4)	7.45 (5)	13.42 (4)	5.63 (5)	10.41 (4)
3	Dr YS Parmer University of Horticulture and Forestry, Solan (Himachal Pradesh)	4.62 (1)	9.50 (3)	1.29 (3)	2.31 (3)	9.05 (2)	15.90 (2)	6.50 (2)	12.05 (2)
4	MPKV Rahuri, Ahmednagar (Maharashtra)	2.90 (12)	6.37 (12)	0.69 (11)	1.66 (12)	4.90 (12)	11.09 (9)	3.26 (12)	7.59 (9)
5	Marathwada Agricultural University, Parbhani (Maharashtra)	4.10 (8)	7.12 (10)	0.94 (8)	2.14 (6)	6.27 (8)	10.14 (10)	4.08 (9)	6.77 (10)
6	Punjab Agricultural University, Ludhiana (Punjab)	3.71 (9)	8.18 (7)	0.79 (9)	2.13 (7)	6.09 (9)	11.90 (8)	4.35 (8)	8.90 (8)
7	Rajendra Agricultural University, Pusa Samastipur (Bihar)	4.33 (6)	8.19 (6)	1.09 (6)	1.96 (10)	8.23 (4)	15.20 (3)	5.66 (4)	10.91 (3)
8	Sher-E-Kashmir University of Agriculture Science and Technology, (Jammu)	4.58 (3)	8.40 (5)	1.40 (1)	2.09 (9)	8.49 (3)	12.12 (7)	6.42 (3)	9.51 (6)
9	Tamil Nadu Agricultural University, Mettupalayam (Tamil Nadu)	4.19 (7)	7.32 (9)	1.28 (4)	1.88 (11)	7.14 (6)	13.18 (6)	4.84 (6)	9.33 (7)
10	UHF RHRS, Jacch, Nurpur, Kangra (Himachal Pradesh)	3.35 (10)	6.50 (11)	0.73 (10)	2.60 (2)	4.92 (11)	8.65 (12)	3.58 (10)	6.65 (11)
11	University of Agriculture Science, Dharwad (Karnataka)	3.28 (11)	7.42 (8)	0.61 (12)	2.12 (8)	5.21 (10)	8.51 (11)	3.44 (11)	6.01 (12)
12	Control	4.42 (5)	9.88 (2)	1.03 (7)	2.67 (1)	6.87 (7)	13.26 (5)	4.79 (7)	9.62 (5)
**Mean**		**4.04**	**8.17**	**1.02**	**2.17**	**7.10**	**12.63**	**5.04**	**9.32**
**LSD**_(P<0.05)_		**0.630**	**1.058**	**0.315**	**0.573**	**1.668**	**2.577**	**1.002**	**1.897**
**SEm**		**0.202**	**0.340**	**0.101**	**0.184**	**0.536**	**0.828**	**0.322**	**0.609**

* Ranks in parentheses

**Table 4:** Change in the ranks* for 54 half-sib families of *Melia azedarach* Linn.

Rank	TH		CBH		BD		DBH	
	Mid-rotation	End-rotation	Mid-rotation	End-rotation	Mid-rotation	End-rotation	Mid-rotation	End-rotation
1	5.64 (12)	11.5 (12)	2.05 (1)	3.15 (37)	15.75 (1)	23.4 (1)	12.17 (1)	18.68 (1)
2	5.55 (1)	11.48 (1)	1.74 (11)	2.76 (13)	13.54 (11)	22.38 (2)	9.92 (11)	17.46 (2)
3	5.14 (7)	11.10 (2)	1.71 (45)	2.68 (39)	10.62 (10)	21.56 (12)	8.54 (10)	14.98 (10)
4	5.11 (11)	11.10 (13)	1.65 (2)	2.67 (54)	10.37 (2)	18.17 (10)	7.98 (2)	14.83 (12)
5	5.10 (8)	10.98 (7)	1.65 (39)	2.61 (29)	10.33 (7)	17.86 (13)	7.77 (7)	13.66 (11)
6	4.82 (10)	10.6 (14)	1.46 (15)	2.6 (49)	10.12 (12)	17.15 (11)	6.98 (43)	13.58 (13)
7	4.72 (46)	10.17 (3)	1.46 (44)	2.55 (11)	9.96 (43)	16.98 (47)	6.54 (45)	12.53 (38)
8	4.70 (40)	10.10 (11)	1.35 (12)	2.54 (16)	8.98 (3)	16.26 (43)	6.50 (37)	11.98 (47)
9	4.69 (43)	9.88 (54)	1.31 (46)	2.48 (38)	8.96 (41)	16.08 (8)	6.43 (3)	11.95 (43)
10	4.62 (13)	9.33 (9)	1.29 (8)	2.46 (15)	8.62 (40)	15.84 (42)	6.33 (40)	11.68 (37)
11	4.55 (45)	9.29 (38)	1.29 (47)	2.42 (1)	8.58 (37)	15.43 (38)	6.30 (12)	11.64 (8)
12	4.54 (9)	9.03 (43)	1.27 (48)	2.42 (48)	8.17 (46)	14.81 (32)	6.094 (44)	11.62 (42)
13	4.54 (14)	9.00 (34)	1.25 (6)	2.41 (23)	8.12 (45)	14.78 (37)	6.08 (25)	11.24 (9)
14	4.52 (2)	9.00 (46)	1.23 (10)	2.41 (34)	8.12 (47)	14.56 (41)	6.05 (46)	11.09 (32)
15	4.51 (25)	8.98 (39)	1.21 (38)	2.39 (31)	7.84 (13)	14.34 (40)	6.04 (41)	10.98 (40)
16	4.50 (41)	8.96 (30)	1.19 (9)	2.39 (50)	7.79 (25)	14.28 (4)	5.82 (9)	10.91 (4)
17	4.43 (37)	8.88 (4)	1.19 (26)	2.38 (8)	7.75 (16)	13.84 (28)	5.77 (6)	10.51 (7)
18	4.42 (54)	8.79 (6)	1.17 (41)	2.38 (9)	7. 72 (44)	13.62 (7)	5.60 (38)	10.43 (15)
19	4.34 (44)	8.75 (29)	1.12 (13)	2.26 (52)	7.50 (42)	13.26 (54)	5.55 (47)	10.42 (6)
20	4.32 (38)	8.50 (45)	1.12 (43)	2.23 (10)	7.44 (14)	13.11 (9)	5.51 (13)	10.2 (41)
21	4.31 (47)	8.42 (16)	1.11 (16)	2.22 (51)	7.29 (6)	12.88 (6)	5.50 (16)	9.98 (16)
22	4.19 (3)	8.38 (41)	1.11 (27)	2.21 (40)	7.25 (8)	12.82 (16)	5.28 (42)	9.88 (28)
23	4.19 (4)	8.33 (37)	1.10 (40)	2.2 (17)	7.23 (9)	12.67 (3)	5.14 (8)	9.68 (39)
24	4.17 (42)	8.17 (50)	1.07 (7)	2.2 (45)	7.17 (38)	12.65 (31)	5.12 (15)	9.63 (46)
25	4.10 (23)	8.08 (17)	1.04 (14)	2.19 (5)	7.12 (4)	12.38 (46)	5.09 (4)	9.62 (54)
26	4.06 (48)	8.04 (40)	1.04 (23)	2.18 (4)	6.87 (54)	12.1 (39)	4.79 (54)	9.55 (3)
27	4.01 (16)	8.00 (42)	1.03 (54)	2.16 (2)	6.46 (27)	12.08 (18)	4.78 (39)	9.37 (25)
28	4.01 (22)	7.96 (32)	1.00 (42)	2.16 (7)	6.46 (51)	11.93 (15)	4.60 (27)	8.91 (31)
29	3.96 (39)	7.96 (35)	0.94 (17)	2.15 (25)	6.38 (31)	11.89 (22)	4.59 (14)	8.80 (26)
30	3.92 (27)	7.92 (25)	0.92 (21)	2.14 (14)	6.29 (23)	11.78 (14)	4.39 (31)	8.38 (45)
31	3.85 (6)	7.88 (10)	0.92 (25)	2.09 (24)	6.25 (39)	11.59 (25)	4.34 (51)	8.23 (5)
32	3.80 (31)	7.88 (47)	0.91 (29)	2.08 (42)	6.21 (29)	11.43 (5)	4.26 (32)	8.20 (21)
33	3.79 (24)	7.83 (33)	0.89 (22)	2.08 (43)	6.16 (48)	11.41 (20)	4.21 (29)	8.08 (27)
34	3.71 (21)	7.75 (27)	0.89 (24)	2.07 (3)	6.06 (15)	11.16 (29)	4.21 (21)	8.08 (44)
35	3.64 (32)	7.75 (31)	0.86 (5)	2.06 (6)	6.00 (32)	10.93 (26)	4.14 (48)	8.03 (34)
36	3.59 (30)	7.61 (28)	0.83 (28)	2.05 (28)	5.79 (30)	10.86 (21)	4.14 (26)	7.93 (17)
37	3.57 (15)	7.54 (8)	0.82 (19)	2.03 (26)	5.71 (21)	10.84 (34)	4.11 (23)	7.93 (19)
38	3.56 (26)	7.43 (26)	0.80 (3)	2.03 (44)	5.67 (33)	10.71 (27)	3.85 (30)	7.87 (18)
39	3.52 (35)	7.40 (53)	0.78 (52)	2.03 (46)	5.54 (24)	10.68 (19)	3.69 (33)	7.86 (14)
40	3.50 (5)	7.33 (5)	0.76 (37)	1.99 (21)	5.46 (22)	10.42 (17)	3.65 (35)	7.83 (29)
41	3.46 (33)	7.30 (51)	0.75 (4)	1.94 (36)	5.37 (26)	10.06 (45)	3.65 (5)	7.42 (35)
42	3.41 (50)	7.25 (21)	0.74 (50)	1.91 (35)	5.16 (5)	9.96 (35)	3.58 (49)	6.73 (36)
43	3.41 (51)	7.17 (24)	0.73 (49)	1.9 (22)	5.07 (35)	9.79 (36)	3.55 (19)	6.68 (48)
44	3.35 (49)	7.13 (36)	0.59 (51)	1.83 (41)	5.04 (36)	9.78 (44)	3.45 (52)	6.65 (49)
45	3.25 (36)	7.08 (44)	0.55 (30)	1.68 (19)	5.01 (50)	9.78 (53)	3.30 (24)	6.58 (22)
46	3.24 (29)	6.94 (19)	0.55 (36)	1.64 (27)	4.96 (19)	9.66 (23)	3.29 (36)	6.43 (23)
47	3.20 (52)	6.88 (15)	0.51 (18)	1.64 (33)	4.92 (49)	9.38 (48)	3.24 (50)	6.24 (20)
48	3.18 (34)	6.79 (52)	0.51 (32)	1.61 (53)	4.92 (53)	8.66 (33)	3.11 (17)	6.23 (52)
49	3.08 (53)	6.75 (23)	0.48 (31)	1.58 (30)	4.79 (18)	8.65 (49)	3.04 (34)	6.22 (50)
50	3.05 (28)	6.75 (48)	0.38 (33)	1.48 (12)	4.69 (20)	8.5 (30)	2.96 (18)	6.02 (33)
51	2.95 (17)	6.63 (22)	0.36 (34)	1.37 (18)	4.46 (52)	8.48 (50)	2.89 (28)	5.95 (53)
52	2.69 (19)	6.50 (49)	0.32 (53)	1.33 (47)	4.37 (28)	8.12 (51)	2.84 (22)	5.87 (30)
53	2.63 (18)	5.08 (18)	0.27 (35)	1.20 (32)	4.37 (34)	7.67 (52)	2.75 (53)	5.64 (51)
54	2.51 (20)	4.48 (20)	0.26 (20)	1.08 (20)	4.33 (17)	7.41 (24)	2.46 (20)	4.80 (24)
**Mean**	**3.99**	**8.26**	**0.99**	**2.12**	**7.06**	**12.72**	**5.04**	**9.45**
**LSD**_(P<0.05)_	**0.941**	**2.159**	**0.545**	**0.860**	**3.009**	**5.245**	**1.839**	**4.150**
**SEm**	**0.333**	**0.763**	**0.193**	**0.304**	**1.064**	**1.854**	**0.650**	**1.467**

* Significant shifts have been indicated

**Note**: Progeny number in parentheses

The analysis of variance showed significant variation among provenances and half-sib families within provenance for all growth parameters. Variability estimates were worked out in terms of phenotypic and genotypic variances (Vg and Vp, respectively) along with the coefficient of variability (phenotypic and genotypic) (Table 2). The analyzed results showed that CBH and DBH were the most important trait with the maximum genotypic coefficient of variation (GCV) for early age followed by BD and TH, whereas, DBH and BD were an important traits with maximum GCV followed by TH and CBH at later ages (Table 2). The heritability and genetic advance of variation for total height (0.55 and 0.98, respectively) and basal diameter (0.54 and 3.07, respectively) provided evidence for the existence of adequate genotypic variations, which lend support for exploitation of genetic variability for further improvement for the species. Similarly, significant variations were recorded earlier for growth parameters among 54 progenies of *M*. *azedarach* [15], among 34 genotypes of *M*. *azedarach* [16], *M*. *volkensii* [17] and 11 progenies in *M*. *azedarach* [18].

In the present study, the broad sense heritability was found moderate to high for all the recorded parameters at an early stage and low to moderate at a later stage. Similarly, Dhillon *et al*. [18] reported moderate to high heritability for height and diameter in *M*. *azedarach* at field condition in initial stage. Low to moderate heritability was also recorded for *Eucalyptus tereticornis* [19]. The coefficient of heritability and genetic gains revealed in the study that satisfactory gains could be obtained with rigorous selection and selection of individuals within the family could further increase the gain proportion at early stage. Similarly, Burton [20] suggested that the study of GCV together with heritability estimates could give the best picture of the success to be achieved through selection. PCV and GCV were relatively higher for all characters at sapling stage and lower at the later stage. This trend can be attributed to the fact that trees contributed maximum energy at the initial stage. The same pattern was by reported by Dhillon *et al*. [18] in *M*. *azedarach*, Kumar *et al*. [19] in *E*. *tereticornis* and Riemenschneider [21] in Jack Pine.

### Growth dynamics

**Fig 3:**
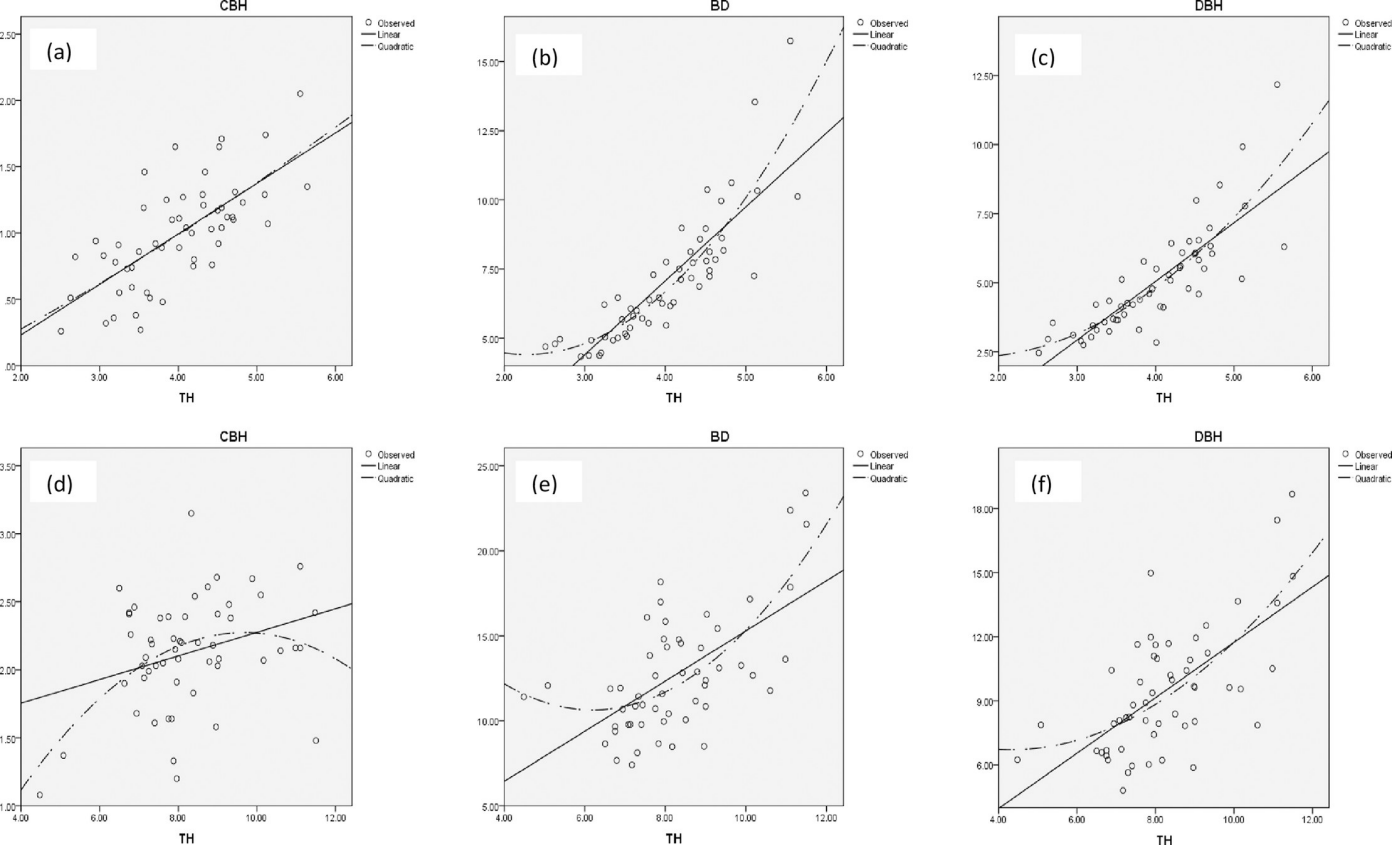
Relationship between values of the tree height function and other growth function (a-c) at mid-rotation and (d-f) at end-rotation age.

There is a contrasting relationship between the tree height and other growth function (The mode of fluctuation) among the families of *M*. *azedarach* (Fig 3). Here, we define the TH-CBH, TH-BD and TH-DBH relationship. First, the linear regression, Y = a_0_ + a_1_X was applied to the Y-X relationship and then quadratic regression was applied (X = value of TH, Y = value of another variable for both rotations). Considerable positive measurements were recorded in BD and DBH in quadratic regression at both rotations (Table 5). A straight line with a positive and negative slope reflects conspicuous differences between growth parameters. A considerable negative measurement was recorded in CBH at fag end of rotation, which may be due to the reduced cultural practices at later stage of trees (Fig 3D). The cultural practices *i*.*e*., planting geometry altered the microclimatic conditions *i*.*e*. moisture, light, nutrients availability etc. in the managed plantation. Similar studies have been carried in block plantation of *M*. *composita* [22] planted at North Indian sub-tropical conditions, 8-years old boundary plantation of *M*. *azedarach* [23] at West Indian semi-arid region of India, mixed block plantation of *Acacia auriculiformis*, *Casuarina equisetifolia* and *Leucaena leuciocephala* [24], paired row block plantation of *Leucaena leucocephala* [25] at Singrauli coalfields, Central India. However, these studies were basically conducted for evaluation of growth characteristics at an early stage of plant growth and studies till maturity are very rarely available due to long rotation period. No systematic study on growth dynamics in any *Melia* species was carried in tropics and this is the first attempt to evaluate the progenies to make use of superior plus trees for harvesting seed for plantations as short term strategy and using the superior progenies/individual by rouging out the inferior ones as long term strategy for harvesting superior seeds for plantations.

**Table 5:** Regression statistics for the growth of 54 half-sib families of *Melia azedarach* Linn.

Period	Regression equation	R^2^	F	P
Mid-rotation	CBH = -0.530 + 0.381 TH	0.482	48.317	< 0.000
	CBH = -0.334 + 0.280 TH + 0.013 TH^2^	0.482	23.732	< 0.000
	BD = -3.619 + 2.673 TH	0.719	132.833	< 0.000
	BD = 8.344–3.470 TH + 0.764 TH^2^	0.765	82.798	< 0.000
	DBH = -3.434 + 2.121 TH	0.673	106.969	< 0.000
	DBH = 3.391–1.384 TH + 0.436 TH^2^	0.695	58.135	< 0.000
End-rotation	CBH = 1.407 + 0.087 TH	0.099	5.715	0.020
	CBH = -1.089 + 0.695 TH—0.036 TH^2^	0.179	5.570	0.006
	BD = 0.538 + 1.475 TH	0.374	31.003	< 0.000
	BD = 23.010–4.000 TH + 0.323 TH^2^	0.458	21.586	< 0.000
	DBH = -1.375 + 1.311 TH	0.429	39.003	< 0.000
	DBH = 9.475–1.333 TH + 0.156 TH^2^	0.457	21.492	< 0.000

The present study deals with the fluctuations in growth with the time. Although the converse is not always true in this case. It can be said that the straight line with positive slope in the Y-TH relationship implies effects among individuals one-sided competition on the growth or suppression in growth rate. Growth regression equations indicated that fluctuations in growth are mostly due to factors other than the effects of one-sided neighborhood competition, e.g. environmental heterogeneity and genetic variation and/or variations in the two-sided competition. From this study, the following results are expected: (i) growth in DBH is affected less by one-sided neighborhood competition than height growth in trees. These results suggested that the growth pattern (*i*.*e*. allocation rate of assimilation) of DBH is genetically determined in a rather strict way. Thus, growth in diameter (*i*.*e*. crown growth type) is important for *M*. *azedarach* as characterized in the introduction and selection of the future breeding program.

### Growth stage selection

**Fig 4:**
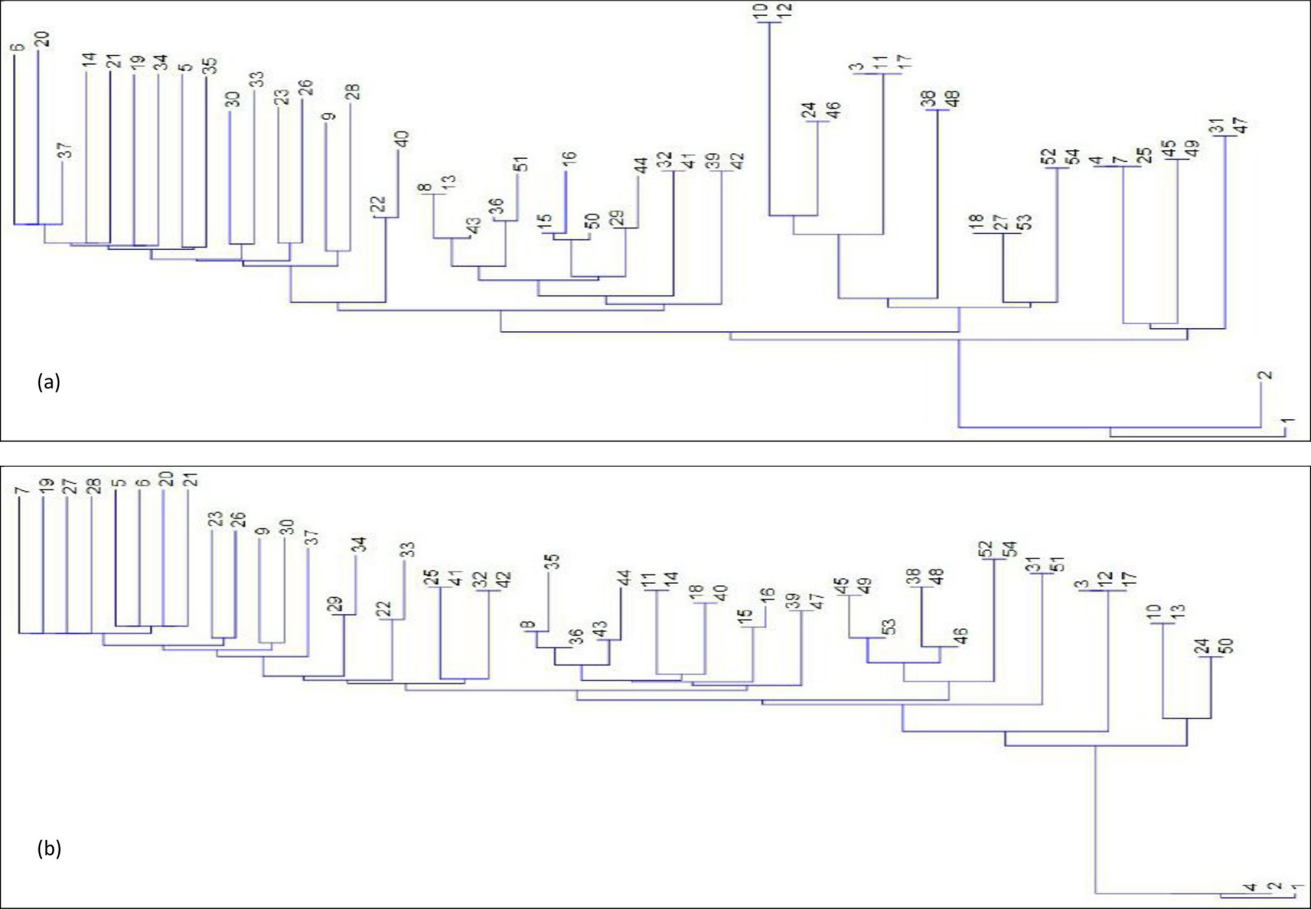
Neighbor-joining clustering with similarity index and out group taxa using morphometric data; (a) mid-rotation, (b) end-rotation.

Perusal of results from the Table 4 revealed that family 12 (check Table 1; Provenance 3- Dr YS Parmar University of Horticulture and Forestry, Solan, Himachal Pradesh) attained maximum TH (5.64 m), whereas, family 1 (Provenance 1- CCS Haryana Agricultural University, Hisar, Haryana) attained maximum CBH (2.05 m), BD (15.75 cm) and DBH (12.17 cm) at mid-rotation. Again family 12 registered maximum TH (11.50 m), family 37 (Provenance 6- Punjab Agricultural University, Ludhiana, Punjab) for CBH (3.15 m), and family 1 for BD (23.40 cm) and DBH (18.68 cm) at rotation age. Family 20 (Table 1; Provenance 4- MPKV Rahuri, Ahmednagar, Maharashtra) was placed at last consistently for TH, CBH and DBH during the entire study period. Similarity index in neighbor-joining cluster analysis revealed that families 1 and 2 (Provenance 1- CCS Haryana Agricultural University, Hisar, Haryana) represented itself as outer taxa at mid-rotation age and end-rotation as well (Fig 4). Family 3 (Provenance 1) originated from CCS Haryana Agricultural University, Hisar, Haryana (Indo-Gangetic central plains, sub-tropical region) and closely related to the family 11 (Provenance 3), originated from Dr YS Parmar University of Horticulture and Forestry, Solan, Himachal Pradesh (Himalayan temperate region) and family 17 (Provenance 4), originated from MPKV Rahuri, Ahmednagar, Maharashtra (Western Ghats, tropical region) at mid-rotation. A similar pattern was observed at end-rotation for family 3.

Thus, families of the same origin were found in the different cluster and the same cluster had the families, which originated from the different geographical regions of India. Seeds procured from CCS Haryana Agricultural University, Hisar, Haryana (Provenance 1; semi-arid region) performed better and maintained its first rank for almost all growth characters and MPKV Rahuri, Ahmednagar, Maharashtra (tropical region) had at last rank (Table 3). First five families for height and basal diameter at mid-rotation were ranked among first ten during end-rotation, which reflects their consistent growth over the years (Table 4). Though, there was swapping of places in different families as well but selection may be preferred at the early stage *i*.*e*. mid-rotation in such fast growing tree species to save time, energy and resources with positive results.

The growth phase generally enters the lag phase from third to fourth years of age in *Melia*, thus heritability estimates carried out after this age would be more reliable. Up to the age of 8-year decreasing trend of heritability for all the traits is though not a positive sign but could not be understood as compared with the results recorded at an early stage of evaluation. Similarly, there are some evidences from several other forest tree species that heritability for growth may increase as genetic test plantations increase with age and that may culminate at about the mid-rotation of the stand [26,27]. Despite the many uncertainties, most tree breeding organizations feel that selections should be made at relatively young age in genetic tests [28] with positive juvenile-mature correlation. Selections can be made most efficiently in young tests if performance of trees at rotation age can be accurately predicted from their performance at younger age. This may be right way to reduce the generation interval in advanced generation tree improvement programme by making selections long before trees have reached rotation age [29].

Families having the same origin had been found distributed in different clusters (Fig 4) that revealed that families with same geographic origin could have undergone changes for different characters under selection, and cultural practices and environmental factors played a vital role in the growth. Additionally, the families originated from same area may have initial genetic differences due to their origin from the seed. The trend in the ranking of different families indicate that the field evaluation and testing of these families need to be carried out for longer duration so that elite germplasm could be screened and deployed for future plantation program. However, the observations of using half rotation for section could be appropriate for long rotation slow growing species like conifers. It is also necessary to examine the trend of all the families over the years so as to group them for different end uses i.e. timber, fuel, fodder, extractives, etc. A consistent pattern of ranking among different sources (average of respective families; Table 3) was observed over the years (top five sources occupied the top position after mid-rotation). However, individuals need to be analyzed for different wood properties before recommending their deployment in future plantation program at large scale. The present evaluation holds good for selection of progenies for fast growth and higher volume production.

## Conclusions

The present study revealed the high variance (σ^2^) and heritability (H^2^) at mid-rotation than end-rotation in *Melia azedarach*. This clearly indicated that environmental factors played an important role in stand structure at later stage. The individual with higher initial growth rate continued with the same trend of relatively higher growth rate at later stage also. TH, CBH, DBH and BD are the function of age and growth pattern in diameter is characterized for the selection of best performing individuals for next generation breeding programmes. It is not essential that the individuals originated in one geographical region showed the same pattern of growth dynamics. These changes may be due to different genetic origin.

## References

[1] Hara T, Kimura M. Kikuzawa K. Growth patterns of tree height and stem diameter in populations of *Abies veitchii*, *A*. *mariesii* and *Betula ermanii*. Journal of Ecology. 1991; 79(4): 1085–1098.

[2] Marks PL. On the relation between extension growth and successional status of deciduous trees of the northeastern United States. Bulletin of the Torrey Botanical Club. 1975; 102: 172–177.

[3] Boojh R, Ramakrishnan PS. Growth strategy of trees related to successional status. I. Architecture and extension growth. Forest Ecology and Management. 1982; 4: 359–374.

[4] Lowman MD. Leaf growth dynamics and herbivory in five species of Australian rain- forest canopy trees. Journal of Ecology. 1992; 80(3): 433–447.

[5] Tomar OS, Minhas PS, Sharma VK, Singh YP, Gupta RK. Performance of 31 tree species and soil conditions in a plantation established with saline irrigation. Forest Ecology and Management. 2003; 177: 333–346.

[6] Chauhan R, Chauhan SK, Saralch HS. Melia azedarach. Bulletin, Department of Forestry and Natural Resources, Punjab Agricultural University, Ludhiana., India. 2008. Pp. 48.

[7] Alche LE, Ferek GA, Meo M, Coto CE, Maier MS. An antiviral meliacarpin from leaves of *Melia azedarach* L. Z Naturforsch C. 2003; 58: 215–219. 12710731 10.1515/znc-2003-3-413

[8] Khangia B. Performance of different forest tree species under agro-climatic condition of Assam. Journal of Agricultural Science Society of North-East India. 1997; 10: 193–196.

[9] Goyal S, Dhul SK, Kapoor KK, Nandal DPS. Soil organic matter and soil microbial properties in *Melia azedarach* plantation in salt affected soil. Indian Journal of Agroforestry. 2001; 3: 130–133.

[10] Marron N, Ceulemans R. Genetic variation of leaf traits related to productivity in a *Populus deltoids* × *Populus nigra*. Canadian Journal of Forest Research. 2006; 36: 390–400.

[11] Hansen JK, Roulund H. Genetic parameters for spiral grain, stem form, Pilodyn and growth in 13 years old clones of Sitka Spruce (*Picea sitchensis* (Bong.) Carr.). Silvae Genetica. 1996; 46: 107–113.

[12] Hai PH, Jansson G, Harwood C, Hannrup B, Thinh HH. Genetic variation in growth, stem straightness and branch thickness in clonal trials of *Acacia auriculiformis* at three contrasting sites in Vietnam. Forest Ecology and Management. 2008; 255: 156–167.

[13] Lush KI. Intra-site correlation and regression of spring on dams as a method of establishing heritability of characters. Proceedings of American Society of Animal Production. 1940; 33: 293–301.

[14] Johnson HW, Robinson HF, Comstock RE. Genotypic and Phenotypic correlations on soyabean and their implications in selection. Agronomy Journal, 1955; 47: 477–483.

[15] Meena H, Kumar A, Sharma R, Chauhan SK, Bhargava KM. Genetic variation for growth and yield parameters in half-sib progenies of *Melia azedarach* Linn. Turkish Journal of Agriculture and Forestry. 2014; 38: 531–539.

[16] Atwal JS. Estimation of genetic variability in dek (Melia azedarach Linn.). M.Sc thesis, Punjab Agricultural University, Ludhiana, India. 2004.

[17] Runo MS, Muluvi GM, Odee DW. Analysis of genetic structure in *Melia volkensii* (Gurke.) populations using random amplified polymorphic DNA. African Journal of Biotechnology. 2004; 3(8): 421–425.

[18] Dhillon GPS, Sidhu DS, Singh B, Singh A. Genetic variation among open pollinated progenies of *Melia azedarach* under nursery and field conditions. Indian Forester. 2009; 135(1): 84–88.

[19] Kumar A, Luna RK, Parveen Kumar V Variability in growth characteristics for different genotypes of *Eucalyptus tereticornis* (SM.) Journal of Forestry Research. 2010; 21(4): 487–491.

[20] Burton GW. Quantitative inheritance in grass. Proceeding of Sixth International Grassland Congress. 1952; 7: 277–283.

[21] Riemenschneider DE. Heritability, age-age correlations, and inferences regarding juvenile selection in Jack Pine. Forest Science. 1988; 34(4): 1076–1082.

[22] Sharma V, Kumar D, Prasad M, Singh C. Effect of Tree Spacing on Growth Performance of Melia composita Willd in Punjab Region of North India. Journal of Agroecology and Natural Resource Management. 2017; 4(4): 298–301.

[23] Roy MM, Pathak PS, Rai AK, Kushwaha D. Tree growth and Biomass production in *Melia azedarach* on farm boundaries in a semi arid region. Indian forester. 2006; 132(1): 105–110.

[24] Shinde PH, Gowade DB. Effect of application of FYM on the availability of nitrogen, potassium and boron in soils. Proceedings of National Seminar on Organic Farming, Pune, India. 1992; Pp. 10–11.

[25] Singh A. Effect of spatial distribution on the growth performance of *Leucaena leucocephala* planted on coal mine spoil. Bulletin of Environment, Pharmacology and Life Sciences. 2012; 1(3): 55–57.

[26] Namkoong G, Usanis RA, Silen RR. Age related variation in genetic control of height growth in Douglas-fir. Theory of Applied Genetics. 1972; 42: 151–159. 10.1007/BF00280791 24430894

[27] Franklin EC. Models relating levels of genetic variance to stand development of four North American conifers. Silvae Genetica. 1979; 28: 207–212.

[28] Lambeth CC. Juvenile-mature correlations in Pinaceae and their implications of early selection. Forest Science. 1980; 26: 571–580.

[29] Zobel BJ, Tabert J. Applied tree improvement 1984; Pp 503 John Wiley & Co., New York, USA.

